# Food Safety: Pop Toxicology?

**Published:** 2006-12

**Authors:** Dinesh C. Sharma

Excessive consumption of carbonated soft drinks has been putatively linked to health effects including dental caries, obesity, and osteoporosis. An August 2006 study by the Centre for Science and Environment (CSE), a private research group in New Delhi, now reiterates another concern first raised three years ago: pesticide contamination.

In *Analysis of Pesticide Residues in Soft Drinks*, the authors report finding an average pesticide content of 11.85 ppb in 11 soft drink brands sold in India by The Coca-Cola Company and PepsiCo. An average of 5.37 ppb lindane, a possible human carcinogen, was detected in the 57 samples analyzed, as was an average of 4.71 ppb chlorpyrifos, a neurotoxicant. Heptachlor was found in 71% of the samples at an average concentration of 0.41 ppb. All these values surpass safe limits of 0.1 ppb for individual pesticides and 0.5 ppb for total pesticides in soft drinks that were proposed by the Bureau of Indian Standards in October 2005. The CSE had released similar findings in 2003.

Industry has contested the findings. Coca-Cola commissioned the analysis of more than 180 samples at Central Science Laboratory (CSL)—a British executive agency—and Vimta Labs in India, and the results showed less than 0.1 ppb of any pesticide. John Gilbert, research director for food at the CSL, says the CSE did not achieve sufficiently good matches between compounds in the samples and known pesticides to confirm identification. “If an identification is not confirmed unequivocally,” he says, “then going on and measuring something of uncertain identity makes no scientific sense.”

In October a panel of India’s health ministry examined the report and rejected the findings, noting that confirmation of pesticide residues in soft drinks by gas chromatography–mass spectrometry (GC-MS) analysis was done for only three samples and did not represent the pesticide residue status of all the 57 test samples. In addition, the CSE improperly used U.S. EPA methodology meant to estimate pesticide concentrations in solids and liquids without validating it for soft drinks. The panel said data received from the CSE relating to “percentage of recovery of different analytes” differed from those in the August 2006 report, raising doubts about the reliability of the results.

Sapna Johnson, lead author of the CSE report, defends the findings, saying they were quantified by two separate detectors. She thinks one reason for the difference in findings of the two studies may be that the CSE used GC-MS only for confirmation of pesticides, whereas the CSL used GC-MS for quantification as well. A CSE statement on the methodology reads, “Test products are fortified with target compounds and are analyzed to demonstrate precision and accuracy of the analyses. Replicate analyses of samples are conducted to confirm that the original results are reproducible. This is how the world tests products. And this is a scientifically valid and socially prudent method.”

“Although the levels of pesticide residues reported [in the CSE] study are unlikely to cause immediate harm to consumers, chronic effects following long-term exposure may occur, particularly in susceptible populations such as children,” says Rolf U. Halden, cofounder of the Johns Hopkins Center for Water and Health. He says the CSE’s data may hint at a larger problem—pesticide-impacted drinking water in the region—and that confirmatory analyses using the best available analytical techniques should be the next step.

## Figures and Tables

**Figure:**
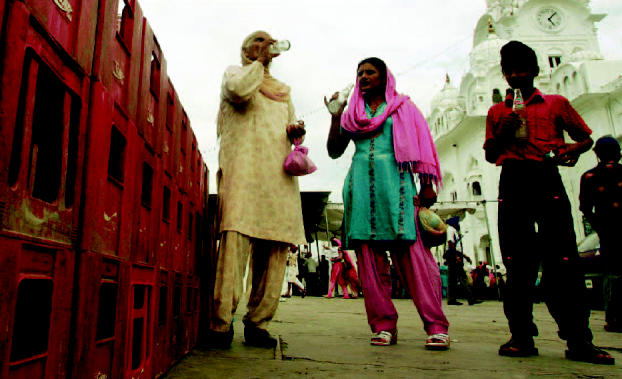
Thirst-quencher threat? People enjoying soft drinks outside the Golden Temple in Amritsar, India. A controversial new report finds that soft drinks made in India may contain high level of pesticides.

